# Prevalence, Morbidity, and Mortality Patterns of Typhoid Ileal Perforation as Seen at the University of Nigeria Teaching Hospital Enugu Nigeria: An 8-year Review

**DOI:** 10.1007/s00268-014-2637-5

**Published:** 2014-05-24

**Authors:** Kenneth Agu, Martin Nzegwu, Emmanuel Obi

**Affiliations:** 1 Department of Surgery, University of Nigeria Teaching Hospital, Enugu, Nigeria; 2 Department of Morbid Anatomy, University of Nigeria Teaching Hospital, Enugu, Nigeria; 3 Department of Community Medicine, University of Nigeria Teaching Hospital, Enugu, Nigeria

## Abstract

**Background:**

Some recent studies have reported a decrease in mortality from typhoid ileal perforation. The present report aims to determine the prevalence, morbidity, and mortality of this disease in patients mostly drawn from a rural area.

**Methods:**

This is a retrospective study of 50 patients treated between January 1999 and December 2007 at the University of Nigeria Teaching Hospital, Enugu, Nigeria. The variables studied included patient demographics, clinical features, intraoperative findings, complications, and mortality. Statistical analysis was done with SPSS version 13.

**Results:**

Of the 50 patients included in the study, 22 were males with the highest rate in patients aged 20 years and younger. Fever was the commonest symptom and at initial presentation, the mean pulse and respiratory rates were significantly higher in the patients who subsequently died than in those who survived (*P* < 0.05). All the perforations occurred in the ileum; 62 % of the patients had solitary perforations, 28 % had double perforations, and 10 % had three or more. Fifty-eight perforations were treated by simple closure in two layers, 4 patients had ileal resection and anastomosis, and 2 underwent right hemicolectomy. The mean interval between operation and death was 1.7 days. The overall mortality rate was 30 %, but among those with three or more perforations, mortality was 100 %.

**Conclusions:**

Typhoid ileal perforation still carries a high mortality especially in rural areas. Those with tachycardia and tachypnea at presentation and those with three or more perforations are at a higher risk of dying from the disease.

## Introduction

Typhoid fever or enteric fever (comprising typhoid and paratyphoid fevers) is a severe infective disease that is endemic in many developing countries [1–3]. Transmission is by the feco-oral route from contamination of food, water, and other drinks by waste from infected patients or carriers. A combination of inadequate sewage disposal, lack of safe drinking water, and unhygienic practices perpetuates the disease in these countries. Perforation of the ileum is the commonest life-threatening complication of typhoid fever [1–5].

The causative organism is a gram-negative motile bacillus with a core lipopolysaccharide antigen, a flagellar antigen, and a virulence antigen. The organism gains access to the host through ingested food or drink and enters the blood stream through the intestinal vessels, having escaped the acidic milieu of the stomach. The illness manifests with various degrees of severity determined by the virulence of the organism and the host’s immunity, among other factors. The major symptoms in uncomplicated cases are fever, abdominal pain, headache, change in bowel habit, anorexia, and nausea [1–4, 6]. Relative bradycardia is commonly reported as an early feature in enteric fever without perforation [7–10]. Involvement of Peyer’s patches in the ileum leads to necrosis and ulceration, which in turn may lead to the two common complications, bleeding and perforation. Classically, perforation occurs in the third week of illness with escalation of morbidity and mortality [11, 12].

Treatment is anchored on resuscitation with fluids, electrolytes, and, sometimes, blood transfusion and broad-spectrum antibiotics [1–4, 13]. At present laparotomy is the preferred modality of treatment with closure of perforation or bowel resection and anastomosis and peritoneal toilet [3, 4, 6, 11, 14–17]. Postoperative complications are common and mortality remains high, most of the patients succumbing to sepsis and multiple organ failure [1–4, 6, 15, 18–21].

Studies on typhoid perforation, both from our center and from other health institutions in Nigeria, have painted a dismal picture of severe morbidity and high mortality. The present study attempted to analyze the current situation with respect to morbidity and mortality of cases seen in our center, which is located in a rural area, and to identify some indicators of mortality.

## Patients and methods

A retrospective search of medical records of patients admitted to our hospital between January 1999 and December 2007 was carried out. A total of 54 patients whose disease was confirmed with blood culture and at laparotomy by ileal perforation were discovered, but four patients were excluded from the study because of insufficient documentation in their case notes. The patients were admitted through the Accident and Emergency Unit of the hospital. All the surgeries were done at the University of Nigeria Teaching Hospital Ituku/Ozalla Enugu, Nigeria and involved two surgery teams. The variables studied included patient demographics and clinical features at presentation, with particular attention to pulse and respiratory rates; findings at operation (number of perforations and distance from the ileocecal valve); postoperative complications, and mortality. Relevant data were extracted from the folders using a proforma and collated. Statistical analysis was done with SPSS version 13. One of the major limitations of the study is its retrospective nature; thus some variables could not be studied due to scanty documentation. The inclusion criterion was the intraoperative finding of the classic antimesenteric ileal perforation with absence of omental reaction. Most of the patients were referred from other hospitals. The details of the treatments they received in those hospitals were not documented. A prospective study is better suited to address questions like the influence of preoperative treatment on the eventual outcome of the disease.

## Results

Of the 50 patients with laparotomy proven perforations, 22 were males. The details of the patient demographics are shown in Table 1. Fever was the commonest symptom, occurring in more than 90 % of the patients. This was followed by abdominal pain, vomiting, and abdominal swelling, in that order (Table 2). There was no record of bradycardia in any of the patients. At initial presentation, the mean pulse and respiratory rates were significantly higher in the patients who subsequently died than in those who survived (*P* < 0.05). The exact time of perforation, and consequently the interval between perforation and operation, could not be determined. The packed cell volume (PCV) which was <30 % in seven patients necessitating preoperative blood transfusion. Five additional patients were transfused postoperatively. There was no derangement of serum electrolytes, urea, and creatinine in any of the patients. Only 18 patients had plain chest and abdominal radiographs, and 11 of them had demonstrable free gas under the diaphragm.

**Table 1:** Demographics and outcome

	Frequency	%
Gender		
Female	28	56.0
Male	22	44.0
Age range, years		
11–20	25	50.0
21–30	14	28.0
31–40	4	8.0
41–50	2	4.0
51–60	5	10.0
Outcome		
Died	15	30.0
Survived	35	70.0

Min = 12 years and max = 58 years, Mean age = 24.5 ± 12.8 years, Median age = 20.5 years

**Table 2:** Symptoms and signs in typhoid ileal perforation

Symptom/sign	*N* (%)
Fever	48 (96)
Abdominal pain	45 (90)
Vomiting	39 (78)
Abdominal swelling	35 (70)
Anorexia	24 (48)
Constipation	24 (48)
Tachypnea	23 (46)
Headache	21 (42)
Diarrhea	14 (28)
Splenomegaly	13 (26)
Hepatomegaly	10 (20)
Jaundice	7 (14)
Melena	4 (8)
Epistaxis	1 (2)
Cough	1 (2)

At presentation all the patients had prompt resuscitation with intravenous saline, nasogastric intubation, and urethral catheterization. All patients also received intravenous metronidazole, ciprofloxacin, or a third-generation cephalosporin. The details and timing of treatment before the patients were referred to our center could not be properly ascertained.

Access was gained into the abdomen through a longitudinal midline incision. All the perforations occurred in the ileum; 62 % of the patients had a solitary perforation, 28 % had double perforations, and 10 % had three or more perforations. Twenty of the perforations (27 %) occurred <15 cm from the ileocecal junction; 46 (62 %), between 15 and 30 cm from it; and 8 (11 %), more than 30 cm from the ileocecal junction. Of the 79 perforations encountered, 58 were treated by simple closure in two layers with Vicryl 2/0 with or without excision of the edges. Four patients who had multiple perforations and unhealthy ileum were treated with ileal resection and anastomosis. Two patients had right hemicolectomy due to diseased ileum and the proximity of the perforations to the ileocecal junction. A summary of the operative techniques employed is shown in Table 3. The surgical wounds were all closed primarily. The commonest complication within the period of admission was surgical site infection (Table 4). The highest number of cases was observed in patients aged 20 years and younger, with the least occurrence in those between 41 and 50 years of age (Table 1). The mean interval between operation and death was 1.7 days (Table 5).

**Table 3:** Surgical treatment of perforations

Surgical treatment	No/% (perforations)
Excision and simple closure	58 (73.4)
Wedge resection	0 (0.0)
Ileostomy	0 (0.0)
Ileal resection and anastomosis	15 (19.0)
Right hemicolectomy	6 (7.6)

**Table 4:** Postoperative complications in typhoid ileal perforation

Complication	Number (%)
Surgical site infection	22 (44)
Hypertrophic scar	12 (24)
Septic shock	10 (20)
Incisional hernia	8 (16)
Enterocutaneous fistula	3 (6)
Re-perforation	3 (6)
Intra-peritoneal abscess	2 (4)
Septic arthritis	1 (2)

**Table 5:** Interval between operation and death, days

Interval	Frequency	%
	*n* = 15	
<one	2	13.3
One	7	46.7
Two	2	13.3
Three	2	13.3
Four	2	13.3

Mean interval = 1.7 ± 1.3 day, Median = 1 day

Patients were admitted for a period of 1–42 days. The mean duration of admission was 17.29 days for survivors and 2.93 days for those who died. The overall mortality rate was 30 %. A greater percentage of females survived, but the difference did not reach statistical significance (Table 4). Two patients died on the operating table, another 7 died within 24 h, and 6 died later than 24 h postoperatively. However, all the deaths occurred within 4 days postoperatively. Among the patients with three or more perforations, the mortality rate was 100 % (Table 6).

**Table 6:** Number of perforations vs outcome cross tabulation

Number of perforations	Outcome		Total
	Died	Survived	*n* = 50 (%)
	*n* = 15 (%)	*n* = 35 (%)	
One	7 (22.6)	24 (77.4)	31 (100.0)
Two	3 (21.4)	11 (78.6)	14 (100.0)
Three	5 (100.0)	0 (0.0)	5 (100.0)

Chi square = 12.969, *P* = 0.002

## Discussion

Typhoid fever and its life-threatening complication ileal perforation is prevalent in our environment as in many developing countries with a lack of safe drinking water and adequate sewage disposal [1–3]. Of the 50 patients we studied, 22 were males (M:F ratio 1:1.3). This is at variance with several similar studies that found a preponderance of males 20–23]. The symptoms and signs (Table 1) are similar to findings by other workers [1–4, 6, 15, 18–21]. Most of the findings of relative bradycardia in some studies involved enteric fever without perforation and were observed early in the illness [7–10]. We did not record any case of relative bradycardia in our patients. This could derive from the fact that, with perforation and peritonitis, other organisms get involved in the infective process and distort the picture. Also, the general late presentation of our patients with perforation may be contributory. We also noted that the mean pulse rate and the mean respiratory rate were significantly higher at initial presentation in patients who subsequently died. This may be an early clinical manifestation of systemic inflammatory response syndrome (SIRS) culminating in septic shock, the commonest cause of death in the patients we studied. Hence these could serve as poor prognostic indicators in patients with typhoid perforation. Other factors including time of presentation, preoperative resuscitation, timing of operation, number of perforations, and extent of fecal peritonitis have been previously identified as prognostic factors [21–24]. Presence of free gas under the diaphragm may not be considered a strong indicator for the diagnosis of typhoid ileal perforation, as only 61 % of the patients had this radiological sign. Lack of some diagnostic tools like laparoscopy could hinder accurate diagnosis in early or doubtful cases. Solitary ileal perforation is the commonest presentation, and in the present study 62 % were solitary. The preponderance of Peyer’s patches, which is the common site of ulceration, might explain the high proportion of perforations occurring in the terminal ileum. However, perforations have been recorded in the upper ileum, the jejunum, and the cecum [18, 25]. Most of the patients, especially those with solitary perforations, were treated with simple closure in two layers after excision of the edges. Several other workers have also employed this surgical option [15, 17, 19, 21, 25]. When the perforations are multiple and close, or when the adjacent ileum is significantly diseased, segmental resection and end-to-end anastomosis is commonly performed [11, 14, 15]. There may be an association between number of perforations and outcome following surgery, as all patients with up to three perforations died, and the observed difference was found to be statistically significant (*P* ≤ 0.05). This association has also been reported by other investigators [6, 22, 24–27]. This finding may be attributed to a higher virulence of the causative organism.

Cecal perforation or ileal perforation close to the ileocecal junction is best treated with right hemicolectomy [18, 25]. One patient in the present study merited such resection.

All the wounds were closed primarily. The surgical site infection rate of 44 % is comparable to the findings of several other workers [24, 27–33]. In our study enterocutaneous fistula was encountered in 6 % of the patients. This is at variance with higher rates found by some other workers [5, 16, 20, 34], and it may be due to the higher resection and anastomosis employed by most of those workers. Septic shock proved a fatal complication in this study; none of the patients who developed it survived. In a similar study done in another teaching hospital in the urban area of Enugu State Nigeria, 86 cases of typhoid perforation were treated over a 2-year period [35]. This might reflect the higher population served by that hospital and probably also higher prevalence of the disease, because of the problems of overcrowding, poor hygiene, and lack of safe drinking water associated with urbanization in many developing countries. This higher incidence in the urban center is, however, associated with an overall lower mortality of 18 %, which may reflect the better care usually obtainable in many hospitals in the urban centers.

The present study also highlights some indicators of mortality, like the presence of three or more perforations, tachycardia, and tachypnea at first presentation. We recorded an overall mortality rate of 30 %, which is similar to reports from other studies [6, 20, 21, 25, 26]. In some more recent studies, however, mortality rates of <20 % have been reported [35, 36]. The relatively higher mortality in this study may be attributable to several factors. Without perforation, patients with typhoid fever are usually managed by general practitioners and internists. Failure to recognize the clinical and laboratory features of perforation and peritonitis generally leads to late referral and poor outcome. The rural setting of the study also provides a possible reason for the higher mortality, which could be related to the poor primary care facilities from which these patients were referred. There was a slight percentage difference in mortality by gender, but this was not statistically significant. There was, however, 100 % mortality in patients with more than two perforations. The mean duration of the hospital stay is significantly higher among the patients who survived than those who died (*P* ≤ 0.05). We also noted that all the deaths occurred within 4 days postoperatively.

Prevention is the ultimate goal for the eradication of typhoid fever. Provision of safe drinking water, proper sewage disposal, and improved personal hygiene are the hallmarks of any preventive intervention. When the disease occurs, mortality could be reduced with early diagnosis, prompt resuscitation, use of potent antibiotics, and emergency operative treatment when perforation and peritonitis supervene.
